# Accurately Identifying *Staphylococcus argenteus* Using Matrix-Assisted Laser Desorption/Ionization Time-of-Flight Mass Spectrometry

**DOI:** 10.3390/diagnostics16152422

**Published:** 2026-07-31

**Authors:** Jia-Ruei Yu, Kai-Wei Huang, Jwu-Ching Shu, Mao-Cheng Ge, Lee-Chung Lin, Tzong-Shi Chiueh, Chih-Pei Lin, Jang-Jih Lu

**Affiliations:** 1 Department of Laboratory Medicine, Chang Gung Memorial Hospital, Linkou Main Branch, Taoyuan City 33305, Taiwan; st33351995@gmail.com (J.-R.Y.); way@cgmh.org.tw (K.-W.H.); jenner7874@cgmh.org.tw (M.-C.G.); leollc@cgmh.org.tw (L.-C.L.); drche0523@cgmh.org.tw (T.-S.C.); 2 Department of Medical Biotechnology and Laboratory Science, College of Medicine, Chang Gung University, Taoyuan City 33302, Taiwan; shujc@mail.cgu.edu.tw; 3 Division of Clinical Pathology, Taipei Tzu Chi Hospital, Buddhist Tzu Chi Medical Foundation, No. 289, Jianguo Rd., Xindian Dist., New Taipei City 23142, Taiwan; cplin@tzuchi.com.tw

**Keywords:** *Staphylococcus argenteus*, MALDI-TOF MS, identification model

## Abstract

**Background/Objectives:** *Staphylococcus argenteus* is a recently recognized member of the *Staphylococcus aureus* complex that is almost identical to *S. aureus* phenotypically and by 16S rRNA gene sequences. Although genomic analyses demonstrate that *S. argenteus* is phylogenetically distinct from *S. aureus*, the two species exhibit more than 90% nucleotide identity and routine identification methods—including routine biochemical assays and matrix-assisted laser desorption/ionization time-of-flight mass spectrometry (MALDI-TOF MS)—cannot reliably distinguish between the two. We develop and validate a MALDI-TOF MS-based model for accurate identification of *S. argenteus*. **Methods:** A multiplex PCR assay targeting *crtM* and *NRPS* genes served as the reference standard. MALDI-TOF MS spectra from 25 *S. argenteus* and 25 methicillin-susceptible *S. aureus* (MSSA) isolates were analyzed using ClinProTools to identify characteristic peaks and develop the identification model. The model was validated using 40 *S. argenteus* and 80 MSSA isolates, then applied to 130 randomly selected clinical isolates. **Results:** Five characteristic peaks—*m*/*z* values 5005, 5285, 5323, 6440, and 6526—were identified. Isolates exhibiting at least 4 of these 5 peaks were classified as *S. argenteus*; those exhibiting fewer than 4 were classified as *S. aureus*. The model achieved 100% specificity and 100% sensitivity in both the development and validation phases. In the clinical application phase, the model correctly classified all isolates, whereas conventional MALDI-TOF MS yielded several misidentifications. **Conclusions:** The identification model, and the simple peak-based rule it is based on, can accurately distinguish *S. argenteus* from MSSA, offering a practical diagnostic tool for clinical microbiology laboratories.

## 1. Introduction

*Staphylococcus argenteus* is a coagulase-positive, Gram-positive coccus and a newly recognized species within the genus *Staphylococcus*. It was first isolated in Australia by McDonald et al. in 2006 [1]. Initially classified as a divergent lineage within the complex, it was established as a separate species in 2015 [2,3,4,5]. While *S. schweitzeri* remains largely restricted to animal hosts and possesses limited clinical relevance in humans [6], *S. argenteus* is a globally distributed pathogen capable of causing a wide spectrum of infections, ranging from skin and soft tissue infections to invasive diseases such as bacteremia and sepsis [7,8,9,10,11,12]. Despite exhibiting relatively low antimicrobial resistance, *S. argenteus* has emerged as a significant concern within the *S. aureus* complex. Its prevalence among isolates previously identified as MSSA has surged—rising from 0.2% in 2000 to 15.2% by 2012 [12]—and it has been associated with higher mortality rates in bloodstream infections compared to typical MSSA [7]. Accurate identification of *S. argenteus* is therefore essential for conducting epidemiological surveillance, monitoring antimicrobial resistance, and understanding its clinical significance.

Distinguishing *S. argenteus* from *S. aureus* presents a significant challenge within routine clinical laboratory settings [5]. From a morphological perspective, *S. argenteus* lacks the golden carotenoid pigment staphyloxanthin [13]. However, colony color alone is an unreliable differentiator, as some *S. aureus* isolates may also appear nonpigmented. Furthermore, both species exhibit remarkably similar profiles in routine biochemical characteristics and hemolytic activity. While most biochemical tests yield nearly identical results, the N-acetylglucosamine acidification test shows a notable divergence; approximately 28% of *S. argenteus* isolates test positive, compared to 98% of *S. aureus* [8]. Nevertheless, such variation remains insufficient to serve as a definitive criterion for clear species differentiation. At the genomic level, *S. argenteus* can be identified by the absence of the *crtM* pigment gene and the presence of a unique 340-bp nonribosomal peptide synthetase (*NRPS*) gene. In contrast, *S. aureus* typically possesses the *crtM* gene and yields a significantly shorter 160-bp *NRPS* PCR product [8]. PCR assays targeting the *crtM* gene and the *NRPS* gene can reliably identify *S. argenteus*; however, most clinical laboratories are not usually equipped to carry out these analyses. Moreover, where such capacity exists, these assays increase considerable cost and turnaround time.

Matrix-assisted laser desorption/ionization time-of-flight mass spectrometry (MALDI-TOF MS) has revolutionized bacterial identification in clinical microbiology laboratories by facilitating rapid and accurate species-level identification. However, even with this method, differentiating between closely related species remains a challenge. Consistent with this limitation, the method often yields ambiguous results for *S. argenteus* and *S. aureus*, both species appearing among the top matches with comparable confidence scores [14]. Several studies have applied advanced computational approaches and developed customized databases to enhance the ability of MALDI-TOF MS to distinguish between closely related bacterial species [12,14]. However, although these strategies improve accuracy, they often require specialized software, extensive datasets, or bioinformatics expertise, and this limits their applicability in routine clinical settings [15,16]. A simple, practical, and reproducible MALDI-TOF MS-based method that can be readily adopted by clinical laboratories without major workflow modifications is therefore a necessity.

The aim of this study is to develop a simple MALDI-TOF MS-based approach that facilitates accurate *S. argenteus* identification in routine clinical practice. By systematically analyzing mass spectral patterns from well-characterized clinical isolates, we identified a small number of distinct peaks that reliably differentiate *S. argenteus* from methicillin- susceptible *S. aureus* (MSSA). We further validated the performance of this peak-based model using independent validation isolates and assessed its applicability across different genetic backgrounds using multilocus sequence typing (MLST) and pulsed-field gel electrophoresis (PFGE). The validation results demonstrate that the MALDI-TOF MS method developed here is a practical solution to an important diagnostic challenge and enables improved recognition of *S. argenteus* in clinical microbiology laboratories.

## 2. Materials and Methods

### 2.1. Bacterial Isolates

The clinical isolates included in this study were procured from the bacterial storage bank of Chang Gung Memorial Hospital and categorized into three functional phases: development, validation, and application. Isolates from all three phases were subcultured on blood agar plates and incubated overnight at 37 °C in 5% CO_2_. Pure cultures were preserved at −70 °C in trypticase soy broth (Difco Laboratories, Detroit, MI, USA) supplemented with 15% glycerol.

The development phase comprised blood culture isolates collected between 2016 and 2018. During this period, *S. argenteus* was not included in the MALDI-TOF database. Because *S. argenteus* is typically methicillin-susceptible [11,12,17,18,19], we retrospectively screened MSSA isolates derived from blood cultures. The isolates were confirmed by multiplex PCR targeting the *crtM* and *NRPS* genes. Ultimately, 25 *S. argenteus* isolates and 25 MSSA isolates were collected in this phase.

The validation phase included blood culture isolates collected between 2021 and 2022. During this period, *S. argenteus* had been incorporated into the MALDI-TOF database. Therefore, 40 *S. argenteus* isolates and 80 MSSA isolates derived from blood cultures were retrospectively collected according to clinical microbiology reports. All isolates were confirmed by multiplex PCR targeting the *crtM* and *NRPS* genes.

In the application phase, conducted from May to December 2023, isolates from all culture sources with ambiguous MALDI-TOF scores meeting the predefined criterion were collected. The identification model was then applied to discriminate between *S. argenteus* and *S. aureus*, and species classification by multiplex PCR was performed as the reference standard. A total of 130 isolates were collected during this phase.

### 2.2. Multiplex PCR-Based Identification of S. argenteus

**Figure 1:**
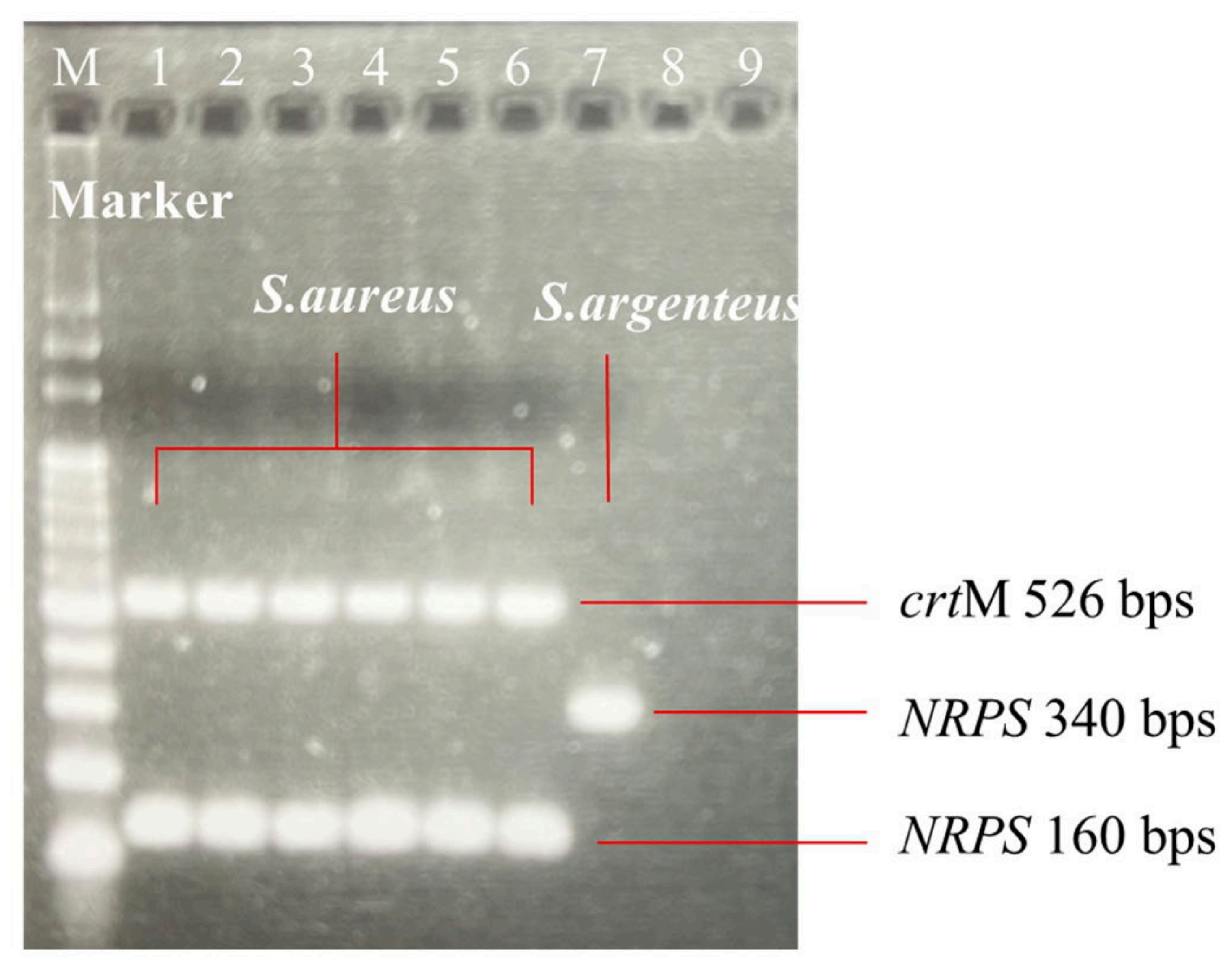
Multiplex PCR differentiating between *S. aureus* and *S. argenteus*. Genomic DNA extracted from clinical isolates was subjected to multiplex PCR targeting the *crtM* and *NRPS* genes. PCR products were separated by agarose gel electrophoresis. Lane M, DNA size marker. Lanes 1–6, *S. aureus* isolates showing bands corresponding to both the 526-bp (*crtM*) and 160-bp (*NRPS*) amplicons. Lane 7, *S. argenteus* isolate showing a band corresponding to only the 340-bp *NRPS* amplicon.

A multiplex PCR assay targeting the *crtM* and *NRPS* genes was established to differentiate *S. argenteus* from *S. aureus*. The *NRPS* primers used in the process were as described by Zhang et al. [8], whereas the *crtM* primers were newly designed in this study (Table 1). Amplification of the *crtM* gene using these newly designed primers generated a 526-bp product in *S. aureus* but not in *S. argenteus* (Figure 1).

To minimize nonspecific amplification, a step-down annealing protocol was employed: 10 cycles with an annealing temperature of 55 °C followed by 20 cycles at 45 °C, optimized for multiplex amplification of both targets. The PCR products were further separated by agarose gel electrophoresis.

As shown in Figure 1, isolates producing both 160-bp (*NRPS*) and 526-bp (*crtM*) amplicons were classified as *S. aureus*, whereas isolates yielding only the 340-bp *NRPS* amplicon were classified as *S. argenteus*. Notably, although *S. schweitzeri* theoretically generates a PCR pattern identical to that of *S. argenteus*, it has minimal clinical relevance in humans; therefore, all clinically isolated strains exhibiting this pattern were regarded as *S. argenteus* in this study.

### 2.3. MALDI-TOF MS Analysis

**Figure 2:**
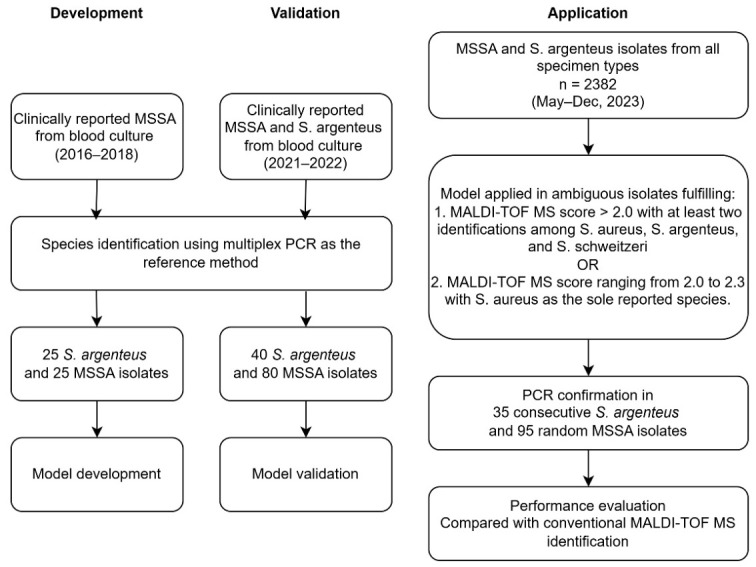
Process flowchart of this study. MSSA, methicillin-susceptible *Staphylococcus aureus*; PCR, polymerase chain reaction; MALDI-TOF MS, matrix-assisted laser desorption/ionization time-of-flight mass spectrometry.

Fresh colonies, from the overnight blood agar cultures of the isolates, were spotted onto a stainless-steel target plate using the direct smear method; formic acid (70%) was subsequently added to each spot, and the plate was air-dried. Each sample spot was overlaid with 1 μL of Bruker HCCA matrix (10 mg/mL), prepared in a standard solvent containing 50% acetonitrile, 47.5% water, and 2.5% trifluoroacetic acid. The spots were air-dried at ambient temperature to ensure uniform crystal formation. Spectra were acquired using a Bruker Microflex LH/SH MALDI-TOF mass spectrometer and analyzed using the Bruker Biotyper software (version 4.1) with the MBT 8468 MSP Library (Bruker Daltonics GmbH, Bremen, Germany) as the reference database. Identification scores ≥2.0 indicated species-level identification. Figure 2 illustrates the process flowchart of this study.

#### 2.3.1. Development

The development phase aims to establish the *S. argenteus* identification model. Twenty-five PCR-confirmed *S. argenteus* isolates and 25 confirmed MSSA isolates were used to train the identification model. The MALDI-TOF MS spectra produced by these isolates were analyzed using ClinProTools 3.0 [20,21,22,23,24,25] and flexAnalysis 3.4 software to identify peaks that differentiate *S. argenteus* from MSSA. Candidate discriminatory peaks were identified by comparing the spectra of *S. argenteus* and MSSA isolates in the development cohort. Peaks that consistently differentiated the two species and showed stable detection across isolates were selected as discriminatory peaks for subsequent analyses. Considering potential instrument-related variation under routine clinical use, a tolerance of ±5 *m*/*z* was applied when defining peak presence (e.g., signals detected within 5000–5010 *m*/*z* were considered positive for the 5005 *m*/*z* peak). The analytical workflow is shown in Figure 1.

#### 2.3.2. Validation

The validation phase aims to acquire the model performance using an independent dataset. 40 PCR-confirmed *S. argenteus* isolates and 80 PCR-confirmed MSSA isolates were collected to validate the MALDI-TOF MS-based identification model. These isolates were run through the newly trained identification model, and the results were then compared with the reference PCR classification (Figure 2).

#### 2.3.3. Application

In the application phase, the *S. argenteus* identification model was applied in the clinical laboratory of Chang Gung Memorial Hospital. The identification model was applied if the isolate met either of the following selection criteria: (1) a MALDI-TOF MS score > 2.0 with at least two potential identifications among *S. aureus*, *S. argenteus*, and *S. schweitzeri*; or (2) a MALDI-TOF MS score ranging from 2.0 to 2.3 with *S. aureus* as the sole reported species.

This phase aims to assess the model feasibility in routine clinical practice. To this end, a total of 130 PCR-confirmed *S. argenteus* and MSSA isolates between May and December 2023 were retrospectively collected. The isolates in this phase were collected from all specimen types. All isolates were subjected to multiplex PCR to serve as the reference method for species confirmation. The sensitivity and specificity of the proposed identification model were subsequently determined. The predictive performance of conventional MALDI-TOF MS identification was also evaluated as a comparator. Under the conventional approach, isolates were classified according to the species with the highest MALDI-TOF MS score (Figure 2).

### 2.4. Phylogenetic Typing

#### 2.4.1. Multilocus Sequence Typing (MLST)

To assess the performance of the identification model across diverse genetic backgrounds, MLST was performed on the 40 *S. argenteus* isolates collected in the second phase. Seven housekeeping genes commonly used in *S. aureus* MLST schemes (*arcC*, *aroE*, *glpF*, *gmk*, *pta*, *tpi*, and *yqiL*) were amplified and subsequently subjected to Sanger sequencing [26,27,28,29,30]. The standard MLST primers were used for *arcC*, *gmk*, and *pta*; however, redesigned primers were used for *aroE*, *glpF*, *tpi*, and *yqiL* owing to the poor or absent amplification associated with the corresponding standard versions used in MLST schemes. Sequence types (STs) were assigned using the *S. aureus* MLST database (https://pubmlst.org/organisms/Staphylococcus-aureus (accessed on 29 April 2026)), with *S. aureus* as the reference species. The phylogenetic relationships and population structures of the STs were investigated using the goeBURST algorithm (version 1.2.1) implemented in PHYLOViZ [31]. Clonal complexes were defined as groups of STs linked by single-locus variants (SLVs). Primer sequences are listed in Table 1.

**Table 1:** Primer sequences used in the multilocus sequence typing in this study.

Primer Name	Primer Sequence (5′ to 3′)	Reference
*crtM*-F	5′ CGTAGAATCATGATGGCGCTTC 3′	Designed in this study
*crtM*-R	5′ TAGCCTGTCTCACTTCGTCCA3′	Designed in this study
*NRPS*-F	5′ TGARWCGACATTACCAGT 3′	[8]
*NRPS*-R	5′ ATWRCRTACATYTCRTTATC 3′	[8]
*arcC*-F	5′ TTGATTCACCAGCGCGTATTGTC 3′	[26]
*arcC*-R	5′ AGGTATCTGCTTCAATCAGCG 3′	[26]
*aroE*-F	5′ CCGTCGATGCATAGTGCAAA 3′	Designed in this study
*aroE*-R	5′ GCCATACCTGCCGGAGTAG 3′	Designed in this study
*glpF*-F	5′ CTTGGAACTGCAATCTTAATCC 3′	Designed in this study
*glpF*-R	5′ AGGTAAAATAGCATGTGCTATTC 3′	Designed in this study
*gmk*-F	5′ ATCGTTTTATCGGGACCATC 3′	[26]
*gmk*-R	5′ TCATTAACTACAACGTAATCGTA 3′	[26]
*pta*-F	5′ GTTAAAATCGTATTACCTGAAGG 3′	[26]
*pta*-R	5′ GACCCTTTTGTTGAAAAGCTTAA 3′	[26]
*tpi*-F	5′ GCGGTTTGGGCAATTTATGC 3′	Designed in this study
*tpi*-R	5′ TCGGCGTCAATACTTCACCT 3′	Designed in this study
*yqiL*-F	5′ CATATAGAACACCAATTGGC 3′	Designed in this study
*yqiL*-R	5′ GAATTGATACCGGAACAATC 3′	Designed in this study

#### 2.4.2. Pulsed-Field Gel Electrophoresis (PFGE)

Following MLST analysis, PFGE was performed on isolates belonging to the predominant sequence type to evaluate genetic diversity within that lineage. In brief, isolates grown for 18–24 h on blood agar plates were resuspended in 250 μL of 0.5× Tris-EDTA buffer and mixed with an equal volume of 1.6% agarose to form plugs. The plugs were placed in microcentrifuge tubes and lysed in lysis buffer (0.006 M Tris-HCl, 0.1 M EDTA, 1 M NaCl, 0.2% deoxycholate, and 0.5% sodium lauryl sulfate) containing lysozyme (1 mg/mL) and lysostaphin (0.05 mg/mL) at 37 °C for at least 6 h. After removing the lysis buffer, the plugs were incubated overnight at 50 °C in ES buffer (0.5 M EDTA, 1% sodium lauryl sulfate) containing 1 mg/mL proteinase K. After removing the ES buffer, the plugs were washed with sterile water at 50 °C for 30 min, followed by three 30-min washes with TE buffer at 37 °C. The plugs were then cut to an appropriate size and digested with *SmaI* restriction enzyme for at least 4 h. The digested plugs were loaded into the wells of a 1.0% agarose gel and separated using a CHEF Mapper XA system (Bio-Rad Laboratories Inc., 94547 Hercules, CA, USA) [32,33,34]. Electrophoresis was performed at 200 V and 14 °C for 22 h, with a pulse angle of 120° and pulse times ranging from 5 to 60 s. PFGE patterns were analyzed using BioNumerics software version 6.5 (BioMérieux, Marcy l’Etoile, France). Isolates with ≥80% similarity were considered the same PFGE type, while those with <80% similarity were classified as different PFGE types.

## 3. Results

### 3.1. MALDI-TOF MS Identification Model: Development

**Figure 3:**
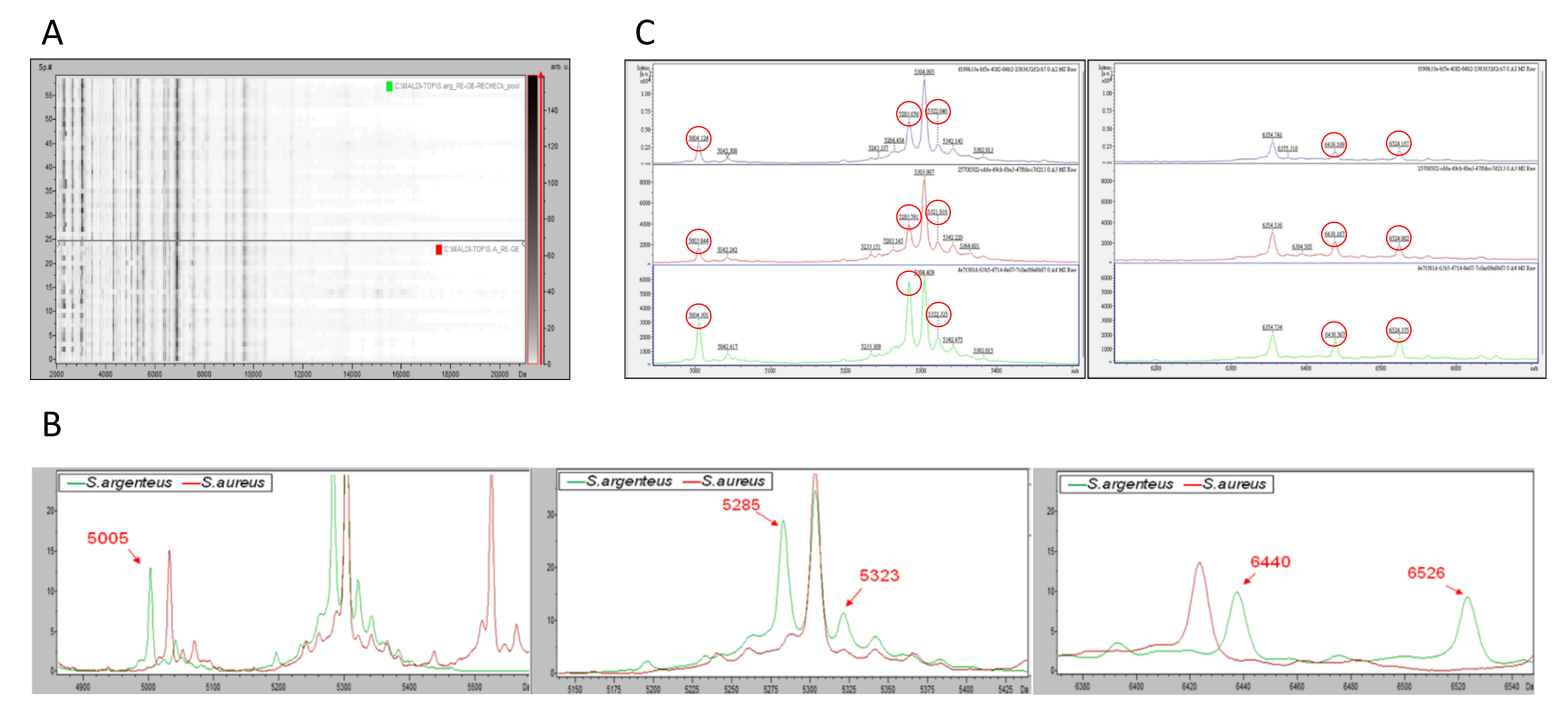
Development of the MALDI-TOF MS identification model. (**A**) ClinProTools visualization displaying the representative MALDI-TOF MS peak profiles of 25 *S. argenteus* isolates (**top**) and 25 MSSA isolates (**bottom**). (**B**) Comparative analysis of MALDI-TOF MS spectra between *S. argenteus* and MSSA, identifying five characteristic peaks—5005, 5285, 5323, 6440, and 6526 *m*/*z*—which serve as key signature markers for species differentiation. (**C**) flexAnalysis results demonstrating that all 25 *S. argenteus* isolates exhibited at least four of the five identified characteristic peaks.

In the development phase, MALDI-TOF MS spectra from 25 PCR-confirmed *S. argenteus* isolates and 25 MSSA isolates were analyzed. Five characteristic peaks were identified and confirmed: 5005 ± 5, 5285 ± 5, 5323 ± 5, 6440 ± 5, and 6526 ± 5 (Figure 3). All 25 *S. argenteus* isolates exhibited four or five of these peaks, whereas all 25 *S. aureus* isolates exhibited fewer than four peaks.

Based on these findings, the MALDI-TOF MS identification criteria were defined as follows: the presence of at least four of the five characteristic peaks was considered indicative of *S. argenteus*, and the presence of less than four peaks was considered indicative of *S. aureus*. The detailed identification rules are summarized in Table 2.

### 3.2. MALDI-TOF MS Identification Model: Validation

All 40 *S. argenteus* isolates and all 80 MSSA PCR-confirmed isolates included in the validation analysis were correctly identified as *S. argenteus* and *S. aureus*, respectively, by the MALDI-TOF MS identification model. The model demonstrated excellent identification accuracy under controlled validation conditions, with 100% sensitivity and specificity (Table 3).

### 3.3. MALDI-TOF MS Identification Model: Clinical Application

Among the 130 isolates analyzed in the application phase, multiplex PCR revealed that the 130 isolates consisted of 35 *S. argenteus* and 95 *S. aureus* isolates. With conventional MALDI-TOF MS identification, 6 of the 35 *S. argenteus* isolates were misidentified as *S. aureus*, and conversely, 1 of the 95 *S. aureus* isolates was misidentified as *S. argenteus*. The sensitivity and specificity for identifying *S. argenteus* were 82.9% and 98.9%, respectively (Table 4).

In contrast, our identification model correctly identified all PCR-confirmed *S. aureus* and *S. argenteus* isolates in the application phase. The model achieved 100% sensitivity and 100% specificity for *S. argenteus* identification (Table 5).

### 3.4. MLST Analysis of S. argenteus

MLST, performed on the 40 *S. argenteus* isolates in the validation set, identified seven sequence types (STs): ST2250, ST1223, ST2198, ST2854, ST3326, ST2793, and ST7299 (Table 6).

ST2250 was the most prevalent ST, followed by ST1223. Three isolates assigned to ST1223 exhibited sequence variation at the *glpF* locus compared with the reference ST1223 *glpF* allele; however, they were still classified as ST1223, based on database analysis. Similarly, two ST2854 isolates were found to harbor a variant glpF locus while maintaining their overall ST2854 classification.

**Figure 4:**
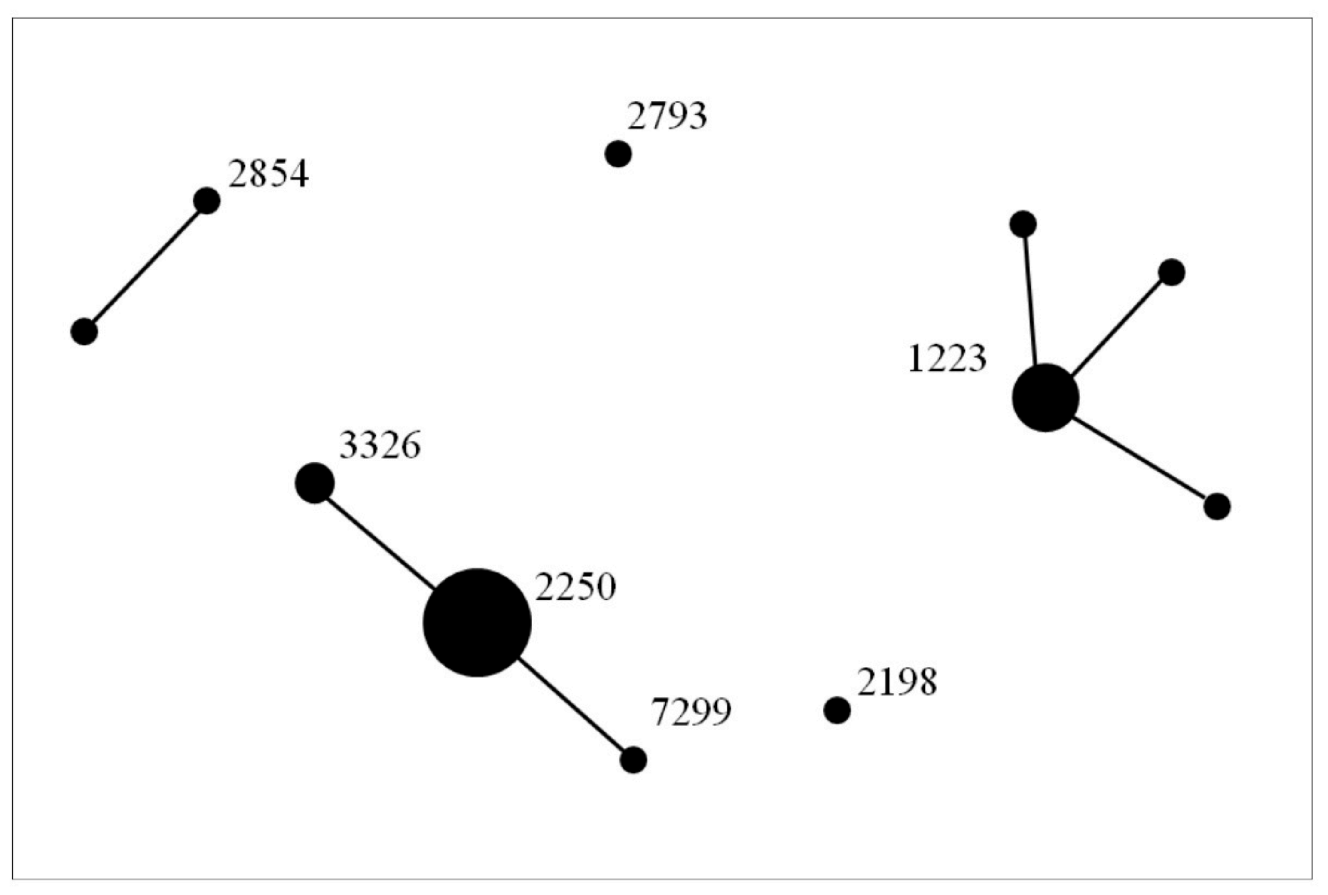
goeBURST analysis of *S. argenteus* isolates based on MLST. Seven sequence types (STs) were identified among the 40 *S. argenteus* isolates in the validation set according to the *S. aureus* MLST scheme as the reference. goeBURST (version 1.2.1; available at www.phyloviz.net) was used to infer clonal relationships between STs. Each node represents an ST, with node size proportional to the number of isolates. Connecting lines indicate single-locus variants (SLVs). ST2250 was the most prevalent ST. ST3326 and ST7299 were SLVs of ST2250, whereas the remaining STs were not closely related within the same clonal complex.

goeBURST analysis identified ST3326 and ST7299 as SLVs of ST2250. The remaining STs differed from ST2250 at three to four loci and from each other at two to three loci, indicating no direct evolutionary relationship (Figure 4).

### 3.5. PFGE Typing of ST2250 S. argenteus Isolates

**Figure 5:**
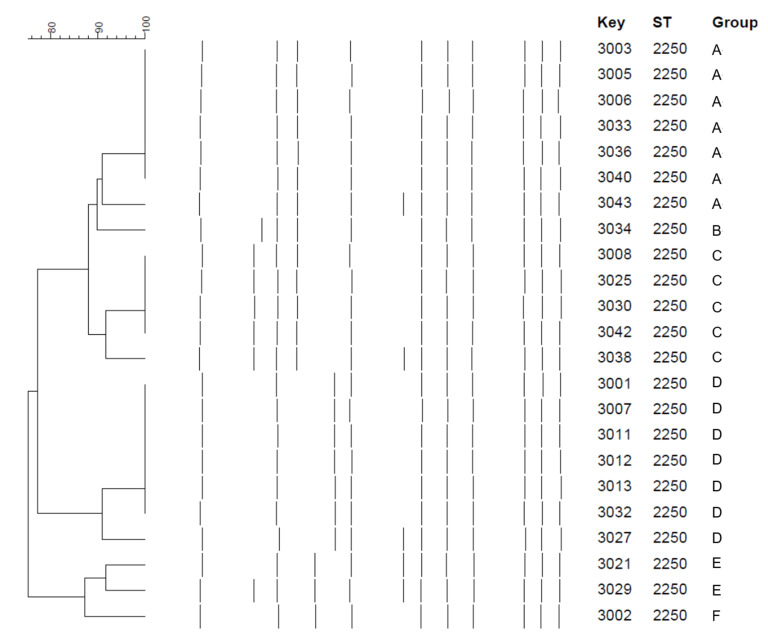
PFGE typing of ST2250 *S. argenteus* isolates. PFGE was performed on 23 *S. argenteus* isolates belonging to ST2250, the most prevalent ST identified by MLST. The dendrogram illustrates genomic relationships among the isolates, revealing six distinct PFGE patterns (A–F) and considerable diversity within the ST2250 lineage. MLST, multilocus sequence typing; PFGE, pulsed-field gel electrophoresis; ST, sequence type.

PFGE of the 23 ST2250 isolates—the most prevalent ST identified—revealed six distinct PFGE patterns (A–F) at a similarity cutoff of 80% (Figure 5). Type A and type D were the most common, each comprising seven isolates, followed by type C (five isolates), type E (two isolates), and types B and F (one isolate each). These results indicate substantial genomic diversity within the ST2250 lineage of *S. argenteus*.

## 4. Discussion

In this study, we developed a simple and accurate MALDI-TOF MS-based model to distinguish *S. argenteus* from MSSA. We identified five characteristic mass spectral peaks (*m*/*z* 5005, 5285, 5323, 6440, and 6526) that differed consistently between the two species. Using the presence of at least four of these five peaks as the criterion for identifying *S. argenteus*, our model achieved 100% sensitivity and specificity in both the development set and an independent validation set. In the clinical application phase, the model correctly identified all PCR-confirmed isolates, whereas the conventional MALDI-TOF MS approach yielded several misidentifications. These findings demonstrate that the peak-based identification criterion can reliably resolve ambiguous MALDI-TOF MS results without the need for additional specialized software or confirmatory wet-laboratory assays.

Differentiating *S. argenteus* from *S. aureus* using standard methods has been challenging because the two species are nearly phenotypically identical. *S. argenteus* lacks the golden carotenoid pigment staphyloxanthin [13], but this difference is subtle and unreliable—some *S. aureus* strains can be nonpigmented. Automated biochemical systems and 16S rRNA sequencing cannot distinguish the two species owing to their high genetic similarity. Even MALDI-TOF MS, which has greatly improved routine bacterial identification, often fails to differentiate these species, frequently returning high-confidence identifications for both in the same analysis. Chen et al. [12] previously developed a MALDI-TOF MS-based classification model using machine learning to differentiate *S. argenteus* from *S. aureus*; however, this model showed limited sensitivity when applied to our dataset. In contrast, our method uses a straightforward peak-based identification rule that can be applied directly in routine clinical laboratories, without the need for complex computational tools—enhancing both accessibility and robustness.

Molecular PCR methods have been proposed as reference standards for identifying *S. argenteus*. Early assays targeted deletions in the *NRPS* gene, while others focused on the *crtM* gene involved in pigment synthesis. Single-target PCR assays, however, may yield indeterminate results due to amplification failure or gene absence. To address these limitations, we adopted a multiplex PCR strategy combining *crtM* and *NRPS* targets; this allows simultaneous confirmation of both species and minimizes false-negative interpretations. This multiplex PCR served as an efficient reference standard during model development. That said, although molecular methods are accurate, they are time-consuming and add cost to routine diagnostic workflows. The MALDI-TOF MS-based identification model, by contrast, leverages spectra already generated during routine identification, allowing immediate resolution of ambiguous results without additional laboratory procedures.

The peak-based discrimination model achieved 100% sensitivity and specificity in both development and validation phases. In the development set, all *S. argenteus* isolates exhibited at least four of the five characteristic peaks, whereas none of the MSSA isolates met this criterion. Chen et al. [12] reported several discriminatory MALDI-TOF MS peaks for identifying the two species, including *m*/*z* 5005.7, 6440.5, 6526.4, and 6635.9 for *S. argenteus* and *m*/*z* 5033.99 for *S. aureus*. In the present study, five peaks—*m*/*z* 5005, 5285, 5323, 6440, and 6526—were used as discriminatory indicators, with isolates classified as *S. argenteus* when at least four of the five peaks were detected. Of these, 3 peaks—*m*/*z* 5005, 6440, and 6526—were previously reported by Chen et al. [12]; *m*/*z* 5285 and 5323 were characteristic peaks newly identified in this study. Lowering the identification threshold from all five peaks to four or more improved sensitivity without compromising specificity. When applied to the independent validation cohort, the model demonstrated complete concordance with PCR reference results. In clinical settings, this approach enables laboratory personnel to resolve ambiguous MALDI-TOF MS identifications by checking for the presence of a small set of predefined peaks, either visually or by simple calculation, without the need for additional procedures. This study is among the first to quantify the extent of *S. argenteus* misclassification in routine clinical MALDI-TOF MS workflows and to provide a practical and implementable solution.

Genetic characterization further supported the broad applicability of the discrimination model. MLST analysis revealed seven distinct STs among the *S. argenteus* isolates, with ST2250 being the predominant lineage, consistent with previous reports [12]. The MALDI-TOF MS identification model correctly identified *S. argenteus* across all STs observed, indicating that its performance is not limited to a specific genetic background. PFGE analysis of ST2250 isolates revealed additional genetic diversity within this lineage, further confirming that the training and validation sets encompassed multiple genetic backgrounds.

Several limitations of this study should be acknowledged. First, this was a single-center study, and the isolate collection may not fully represent the global genetic diversity of *S. argenteus*. The predominant sequence types may vary across geographic regions, and the performance of the proposed model may therefore differ in other genetic and geographic settings. Multicenter and international validation studies involving genetically diverse isolates are warranted to further evaluate the generalizability and robustness of the proposed model. Second, the identification model was developed and tested using a single MALDI-TOF MS platform, specifically the Bruker Microflex LH/SH system with the software and database configurations used in this study. The model performance may vary across different MALDI-TOF MS systems and configurations. Therefore, independent validation is necessary before implementation on other MALDI-TOF MS systems or configurations to confirm the reproducibility and performance of the proposed model. Third, whole-genome sequencing was not used as a reference standard, which represents a methodological limitation given its superior resolution for species confirmation. Therefore, the performance of the proposed model should be interpreted as its ability to identify isolates confirmed as *S. argenteus* or MSSA by the multiplex PCR reference method, rather than as definitive species identification based on a gold-standard reference method. In conclusion, future multicenter studies incorporating larger and more diverse isolate collections and multiple MALDI-TOF systems will be necessary to further validate and refine this approach. Despite these limitations, the findings of this study demonstrate that a simple, peak-based MALDI-TOF MS identification model can substantially improve the accurate identification of *S. argenteus* in routine clinical microbiology laboratories.

## 5. Conclusions

This study developed a simple MALDI-TOF MS peak-based identification model to distinguish *S. argenteus* from MSSA. The model achieved 100% sensitivity and specificity in both validation and clinical application phases, suggesting it is a practical diagnostic tool for clinical microbiology laboratories.

## Figures and Tables

**Table 2:** Identification rules for MALDI-TOF MS-based identification of *S. argenteus*.

Peak	5005	5285	5323	6440	6526
Tolerance (*m*/*z*)	±5	±5	±5	±5	±5
*S. argenteus*	≥4 peaks in the 5 peaks				
*S. aureus*	<4 peaks in the 5 peaks				

**Table 3:** Validation results of the MALDI-TOF MS-based identification model for identification of *S. argenteus*.

		Reference Standard (PCR)		
		*S. argenteus*	*S. aureus*	Total
Identification model	*S. argenteus*	40	0	40
	*S. aureus*	0	80	80
	Total	40	80	120

**Table 4:** Performance of conventional MALDI-TOF MS in identifying *S. argenteus* in the clinical applicability cohort.

		Reference Standard (PCR)		
		*S. argenteus*	*S. aureus*	Total
Prediction by conventional MALDI-TOF MS	*S. argenteus*	29	1	30
	*S. aureus*	6	94	100
	Total	35	95	130

**Table 5:** Performance of the identification model in identifying *S. argenteus* in the clinical applicability cohort.

		Reference Standard (PCR)		
		*S. argenteus*	*S. aureus*	Total
Prediction by our identification model	*S. argenteus*	35	0	35
	*S. aureus*	0	95	95
	Total	35	95	130

**Table 6:** MLST results of *S. argenteus* isolates.

arcC	aroE	glpF	gmk	pta	tpi	yqiL	ST Type	Isolate Numbers (%)
151	325	215	34	175	180	169	2250	23 (57.5%)
151	187	20	101	145	150	131	1223	9 (22.5%)
151	187	270	101	145	150	131	1223 *	
151	187	321	101	145	150	131	1223 *	
151	187	43	101	145	150	131	1223 *	
36	269	43	34	107	116	105	2198	2 (5%)
36	406	321	34	287	52	302	2854	2 (5%)
36	406	270	34	287	52	302	2854 *	
151	325	448	34	175	180	169	3326	2 (5%)
151	406	43	34	256	261	323	2793	1 (2.5%)
151	325	43	34	175	180	169	7299	1 (2.5%)

^*^ These isolates differed from the canonical Staphylococcus aureus MLST profiles at one or more loci. Therefore, the closest matching STs generated by the MLST database were assigned. MLST, multilocus sequence typing; ST, sequence type.

## Data Availability

The data presented in this study are available on request from the corresponding author. The data are not publicly available due to privacy.

## References

[1] McDonald M., Dougall A., Holt D., Huygens F., Oppedisano F., Giffard P.M., Inman-Bamber J., Stephens A.J., Towers R., Carapetis J.R. (2006). Use of a single-nucleotide polymorphism genotyping system to demonstrate the unique epidemiology of methicillin-resistant *Staphylococcus aureus* in remote aboriginal communities. J. Clin. Microbiol..

[2] Ng J.W., Holt D.C., Lilliebridge R.A., Stephens A.J., Huygens F., Tong S.Y., Currie B.J., Giffard P.M. (2009). Phylogenetically distinct *Staphylococcus aureus* lineage prevalent among indigenous communities in northern Australia. J. Clin. Microbiol..

[3] Ruimy R., Armand-Lefevre L., Barbier F., Ruppé E., Cocojaru R., Mesli Y., Maiga A., Benkalfat M., Benchouk S., Hassaine H. (2009). Comparisons between geographically diverse samples of carried *Staphylococcus aureus*. J. Bacteriol..

[4] Tong S.Y., Lilliebridge R.A., Bishop E.J., Cheng A.C., Holt D.C., McDonald M.I., Giffard P.M., Currie B.J., Boutlis C.S. (2010). Clinical correlates of Panton-Valentine leukocidin (PVL), PVL isoforms, and clonal complex in the *Staphylococcus aureus* population of Northern Australia. J. Infect. Dis..

[5] Tong S.Y., Schaumburg F., Ellington M.J., Corander J., Pichon B., Leendertz F., Bentley S.D., Parkhill J., Holt D.C., Peters G. (2015). Novel staphylococcal species that form part of a *Staphylococcus aureus*-related complex: The non-pigmented *Staphylococcus argenteus* sp. nov. and the non-human primate-associated *Staphylococcus schweitzeri* sp. nov. Int. J. Syst. Evol. Microbiol..

[6] Akoua-Koffi C., Kacou N’Douba A., Djaman J.A., Herrmann M., Schaumburg F., Niemann S. (2022). *Staphylococcus schweitzeri*—An Emerging One Health Pathogen?. Microorganisms.

[7] Chen S.-Y., Lee H., Wang X.-M., Lee T.-F., Liao C.-H., Teng L.-J., Hsueh P.-R. (2018). High mortality impact of *Staphylococcus argenteus* on patients with community-onset staphylococcal bacteraemia. Int. J. Antimicrob. Agents.

[8] Zhang D.-F., Xu X., Song Q., Bai Y., Zhang Y., Song M., Shi C., Shi X. (2016). Identification of *Staphylococcus argenteus* in Eastern China based on a nonribosomal peptide synthetase (NRPS) gene. Future Microbiol..

[9] Thaipadungpanit J., Amornchai P., Nickerson E.K., Wongsuvan G., Wuthiekanun V., Limmathurotsakul D., Peacock S.J. (2015). Clinical and molecular epidemiology of *Staphylococcus argenteus* infections in Thailand. J. Clin. Microbiol..

[10] Hansen T.A., Bartels M.D., Høgh S.V., Dons L.E., Pedersen M., Jensen T.G., Kemp M., Skov M.N., Gumpert H., Worning P. (2017). Whole genome sequencing of Danish *Staphylococcus argenteus* reveals a genetically diverse collection with clear separation from *Staphylococcus aureus*. Front. Microbiol..

[11] Kitagawa H., Ohge H., Hisatsune J., Masuda K., Aziz F., Hara T., Kuroo Y., Sugai M. (2020). Low incidence of *Staphylococcus argenteus* bacteremia in Hiroshima, Japan. J. Infect. Chemother..

[12] Chen S.-Y., Lee H., Teng S.-H., Wang X.-M., Lee T.-F., Huang Y.-C., Liao C.-H., Teng L.-J., Hsueh P.-R. (2018). Accurate differentiation of novel *Staphylococcus argenteus* from *Staphylococcus aureus* using MALDI-TOF MS. Future Microbiol..

[13] Holt D.C., Holden M.T., Tong S.Y., Castillo-Ramirez S., Clarke L., Quail M.A., Currie B.J., Parkhill J., Bentley S.D., Feil E.J. (2011). A very early-branching *Staphylococcus aureus* lineage lacking the carotenoid pigment staphyloxanthin. Genome Biol. Evol..

[14] Eshaghi A., Bommersbach C., Zittermann S., Burnham C.-A.D., Patel R., Schuetz A.N., Patel S.N., Kus J.V. (2021). Phenotypic and genomic profiling of *Staphylococcus argenteus* in Canada and the United States and recommendations for clinical result reporting. J. Clin. Microbiol..

[15] Wang H.-Y., Chung C.-R., Tseng Y.-J., Yu J.-R., Chen C.-J., Wu M.-H., Lin T.-W., Huang W.-T., Liu T.-P., Lee T.-Y. (2021). Extensive validation and prospective observation of the impact of an AI-based rapid antibiotics susceptibility prediction platform in multiple medical centers. medRxiv.

[16] Yu J.-R., Chen C.-H., Huang T.-W., Lu J.-J., Chung C.-R., Lin T.-W., Wu M.-H., Tseng Y.-J., Wang H.-Y. (2022). Energy efficiency of inference algorithms for clinical laboratory data sets: Green artificial intelligence study. J. Med. Internet Res..

[17] Chantratita N., Wikraiphat C., Tandhavanant S., Wongsuvan G., Ariyaprasert P., Suntornsut P., Thaipadungpanit J., Teerawattanasook N., Jutrakul Y., Srisurat N. (2016). Comparison of community-onset *Staphylococcus argenteus* and *Staphylococcus aureus* sepsis in Thailand: A prospective multicentre observational study. Clin. Microbiol. Infect..

[18] Rong D., Liu Z., Huang J., Zhang F., Wu Q., Dai J., Li Y., Zhao M., Li Q., Zhang J. (2023). Prevalence and characterization of *Staphylococcus aureus* and *Staphylococcus argenteus* isolated from rice and flour products in Guangdong, China. Int. J. Food Microbiol..

[19] Hao S., Abdelghany M., Lyden A., Sit R., Tan M., Tato C.M., DeRisi J.L., Miller S., Doernberg S.B., Langelier C. (2020). Genomic profiling of evolving daptomycin resistance in a patient with recurrent *Staphylococcus argenteus* sepsis. Antimicrob. Agents Chemother..

[20] Huang C.-H., Huang L. (2018). Rapid species-and subspecies-specific level classification and identification of Lactobacillus casei group members using MALDI Biotyper combined with ClinProTools. J. Dairy Sci..

[21] Wang H.-Y., Lien F., Liu T.-P., Chen C.-H., Chen C.-J., Lu J.-J. (2018). Application of a MALDI-TOF analysis platform (ClinProTools) for rapid and preliminary report of MRSA sequence types in Taiwan. PeerJ.

[22] Huang Y., Xu Y., Huang Y., Sun F., Tian H., Hu N., Shi L., Hua H. (2019). Identification of newly developed advanced schistosomiasis with MALDI-TOF mass spectrometry and ClinProTools analysis. Parasite.

[23] Kubo Y., Ueda O., Nagamitsu S., Yamanishi H., Nakamura A., Komatsu M. (2021). Novel strategy of rapid typing of Shiga toxin-producing *Escherichia coli* using MALDI Biotyper and ClinProTools analysis. J. Infect. Chemother..

[24] Nakayama A., Morinaga Y., Izuno R., Morikane K., Yanagihara K. (2024). Evaluation of MALDI-TOF mass spectrometry coupled with ClinProTools as a rapid tool for toxin-producing *Clostridioides difficile*. J. Infect. Chemother..

[25] Zhang T., Ding J., Rao X., Yu J., Chu M., Ren W., Wang L., Xue W. (2015). Analysis of methicillin-resistant *Staphylococcus aureus* major clonal lineages by matrix-assisted laser desorption ionization–time of flight mass spectrometry (MALDI–TOF MS). J. Microbiol. Methods.

[26] Enright M.C., Day N.P., Davies C.E., Peacock S.J., Spratt B.G. (2000). Multilocus sequence typing for characterization of methicillin-resistant and methicillin-susceptible clones of *Staphylococcus aureus*. J. Clin. Microbiol..

[27] Feil E.J., Cooper J.E., Grundmann H., Robinson D.A., Enright M.C., Berendt T., Peacock S.J., Smith J.M., Murphy M., Spratt B.G. (2003). How clonal is *Staphylococcus aureus*?. J. Bacteriol..

[28] Park K.-H., Greenwood-Quaintance K.E., Uhl J.R., Cunningham S.A., Chia N., Jeraldo P.R., Sampathkumar P., Nelson H., Patel R. (2017). Molecular epidemiology of *Staphylococcus aureus* bacteremia in a single large Minnesota medical center in 2015 as assessed using MLST, core genome MLST and *spa* typing. PLoS ONE.

[29] Hsu J.-C., Wan T.-W., Lee H., Wang X.-M., Lin Y.-T., Jung C.-J., Lee T.-F., Hsueh P.-R., Teng L.-J. (2020). Heterogeneity of molecular characteristics among *Staphylococcus argenteus* clinical isolates (ST2250, ST2793, ST1223, and ST2198) in northern Taiwan. Microorganisms.

[30] Chen J.H., Leung H.-Y., Wong C.M., Yuen K.-Y., Cheng V.C. (2023). Prevalence and characteristics of invasive *Staphylococcus argenteus* among patients with bacteremia in Hong Kong. Microorganisms.

[31] Francisco A.P., Bugalho M., Ramirez M., Carriço J.A. (2009). Global optimal eBURST analysis of multilocus typing data using a graphic matroid approach. BMC Bioinform..

[32] Rocha Balzan L.D.L., Rossato A.M., Riche C.V.W., Cantarelli V.V., D’Azevedo P.A., Valério de Lima A., Rodrigues B., Franca e Silva I.L.A., Dias C.A.G., Sampaio J.L.M. (2023). *Staphylococcus argenteus* Infections, Brazil. Microbiol. Spectr..

[33] Bank L.E., Bosch T., Schouls L.M., Weersink A.J., Witteveen S., Wolffs P.F., Nijhuis R.H. (2021). Methicillin-resistant *Staphylococcus argenteus* in the Netherlands: Not a new arrival. Eur. J. Clin. Microbiol. Infect. Dis..

[34] Witteveen S., Hendrickx A.P., de Haan A., Notermans D.W., Landman F., van Santen-Verheuvel M.G., de Greeff S.C., Kuijper E.J., van Maarseveen N.M., Vainio S. (2022). Genetic characteristics of methicillin-resistant *Staphylococcus argenteus* isolates collected in the dutch national MRSA surveillance from 2008 to 2021. Microbiol. Spectr..

